# Snatches of time. Fragments

**DOI:** 10.1093/brain/awaf166

**Published:** 2025-06-03

**Authors:** Matthew Butler

**Affiliations:** King’s College London, London, UK

## Abstract

Matt Butler, runner up in the Brain Essay Competition 2024, considers what happens when memories fragment and certainties fade in this fictional tale of a professor of literature who loses her grasp on time.

***This essay is the fictional tale of a professor of literature who loses her grasp on time as it begins to slip backwards from her. It imagines what happens once memories fragment and certainties fade. It is a story of nostalgia, dissolution, and dementia.***

A snowflake, cold upon her cheek. A shudder, and she is back atop the mountain.

They ride the funicular up to the old sanitorium. Suitcases unpacked, sheathed in woollen garments, they recline on balcony chairs. The craggy white peaks of the Swiss Alps glisten in the low sunshine. She pulls her blanket tighter and reaches out a hand to her husband beside her.

A paperback slides from her lap to the floor.

He picks it up and thumbs it. ‘What are you reading?’

‘Marcel Proust,’ she replies, ‘*In Search of Lost Time*. Or as some cruder translations would have it, *Remembrance of Things Past*.’ She makes mocking quotation marks in the air.

‘Sounds exactly like something you would read,’ he smiles.

‘It’s for next term, my module on Time in Literature.’

‘I thought time was the domain of serious scientists like me?’ he says, half-mockingly.

‘Nothing like fiction to manifest the slippery, wobbly form of time. Proust most famously, I suppose …’

‘You know,’ he continues, ‘back in the Twenties, a novelist … or maybe a philosopher … Anyway, some smart aleck challenged Albert Einstein to a public debate about special relativity. Imagine having the gall to do that! He argued science should *stay out of the way of time*, because it can never be pinned down by numbers; only by intuition, or something … can’t say I agree, I think we have to scrutinize time objectively, probe its inner workings, dismantle it piece by piece through the scientific method.’

‘You remind me,’ she replies with a smirk, ‘of a character in *Catch-22* who tries to prolong his existence; not in the conventional way of healthy eating, exercise, et cetera. Instead, he attempts, in earnest, to stretch out the duration of his life to its maximal limit. He tries to warp subjective time. He starts by undertaking the most menial task he can possibly think of: taking apart oil lamps, arranging the pieces, and putting them back together again.’

He turns to her, eyes creased at the corners in the way she has always loved.

‘You might say he was a cultivator, nay! a connoisseur, of boredom, Mike. And hearing you talk about physics again is simply life imitating art …’

He picks up a pack of cards and goes as if to throw it at her, he laughs and she laughs back, trying to hold in the smoke from the spliff that someone just passed over. The air is thick with the haze of life. From the stage, the peal of a hundred bells. The crowd call out for Pink Floyd to start their set.

The ticking of a clock. The jangle of a guitar. This next song’s called *Time*.

‘They wrote this album about their ex-singer, you know? He went a bit loony, had a breakdown, lost his mind, the poor lad.’

She looks down at the floor, at his flares. The music begins.

She turns to meet his gaze, the two intoxicated by existence, enveloped by the now. Their souls vibrate to the tune of the music, the grass, their love.

He leans in, his long hair falling over hers, intertwining. They kiss, as sumptuous as the sustained chords.

*… you are young, and life is long, and there is time to kill today …*

She could have stayed in that moment forever.

*… you run and you run to catch up with the sun, but it's sinking …*

She runs up the stairs, clutching her bag and shuffling past crossed legs which reluctantly let her past. The lecture has already begun. At the front, bespectacled, he takes a sip of water and places a slide on the projector. On the screen, arrows point higgledy-piggledy amidst a bustling crowd of circles.

‘You are nearing the end of your first fundamentals module,’ he begins. She sticks out, a trespasser amongst the undergraduates, but she is captivated by the way he holds the room, his shy confidence. His students tap their pencils in thought, scribble some notes. Recording what he says. Putting words to memory.

‘… time is fascinating,’ he continues, ‘for many reasons, but not least because almost every law of physics does not require it. We live in a time-symmetrical universe, for the most part.’

A pause for effect.

‘Practically all of our equations work just as well if time runs forwards or backwards. It doesn’t matter. Time is unnecessary. That is, it is unnecessary to all quantities but one.’

He points to a keen hand at the front.

‘Correct, entropy,’ he nods.

‘The fact that heat should always, on average, move from a hotter to a colder place over time. Entropy is the only thing that really tenses the bow that fires the arrow of time. Indeed, entropy may be the only reason why we have time at all.’

Murmurings around the room, and a hand from the back shoots up to ask for another glass of wine, a drop of crimson soaking into the kitchen table. They are sat together on battered stools, gossiping, laughing, gambling.

That night, the night when they first met, time stood still.

She lights a cigarette and watches him shuffle the new deck, the reds and the blacks slowly blurring into each other. She sighs contentedly, drunk in wonder at how they have drifted into each other’s arms amidst the chaos and uncertainty. He lays out the deck, gently brushing her hand as he does so. The precise configuration of each and every shuffle has never happened before in the history of the universe.

It is late. They are chatting, mellow from the merlot. ‘Could it be that time is only a product of our brains? That time is only necessary for our own experience? So, so’—she gesticulates with her glass—‘time, well it may not be an actual thing? It might just be constructed by the mind? So, when the mind goes, time does too?’

‘Hippie!’ Her flatmate sniggers. ‘You sound like someone who’s just dropped acid, maaan … I had a friend once who tripped so hard she said time was just a balloon that she could inflate or deflate at her whim.’

‘I don’t know, sweetheart,’ interjects a foppish looking friend-of-a-friend called Mike, who looks deeply into her, ‘the physical constraints on time aren’t all that robust, time stands still at the speed of light; perhaps the arrow of time shoots less true than we’d assume.’

‘*Time’s Arrow*, students, by Martin Amis: the next book on your reading list. A masterpiece in reverse chronology. The protagonist, a German doctor, propelled backwards from his future towards the atrocities he committed in concentration camps. Heavy going, but needs to be read, never to be forgotten …’

A doctor approaches, chatting to a colleague, halting in the doorway.

She turns onto her side, the bed itchy and uncomfortable. It smells musty. Her thin, frail arms. Delicate, blue-ribboned paper.

‘Where is Michael?’ she asks.

The disorientating throng around her. *Where is Michael?* She twists towards Wembley Park station. Cheers and hollers, the deafening noise of a football rattle. She cannot find him.

Her student’s essay; *1966 And All That: Collective Memory and the Modern English National Identity*. Newspaper clippings about that day, a famous World Cup victory. She cried whilst marking it, an unintentional reminder of him.

She was there. He *was* there.

A memory as bright as the flashbulbs of the baying press.

‘Please, where is Mike? I’ve lost him! Help me …’ her mind whirls in frantic circles.

… a disrupted phenomenology of time is seen in many neuropsychiatric disorders … in bipolar mania, for example, time perception speeds up to a frenzy … we’re not sure of the mechanism, there’s no time organ in the brain … but the sense of time passing is fundamental to our experience, it underpins our striving, sets out our goals ahead of us …

*Some people are on the pitch! They think it’s all over!* A clipped and crackling voice announces on the wireless. A goal in the final moment.

‘Who remembers that day?’ A man in a freshly ironed white polo shirt and a name badge asks. ‘It’s certainly one to tell the children about.’

They never had children. Never found the time.

… a confusion in ordering past events … inconsistencies in time estimation … it may be due to disruptions in attention, shattered working memory … things fall apart, the hippocampi atrophy, perhaps there is a loss of time cells which encode temporal experiences … but a relative preservation of autobiographical memories … at least until the latter stages …

He flew eastwards to a conference on artificial intelligence. He was concerned, sensitive soul, about the ramifications of unchecked progress. Something about quantum, memory, computations.

On the news, an attack. An explosion. A disintegration.

She calls out to him.

… little islands of remembrance persist, often older, more emotional memories … entropy and disorder are inexorable; life is but a brief fight against these forces. Not even the brain, the most complex thing there ever was, can escape the Way of All Things. In disorders like this we simply see the cruel unravelling happening slowly, tragically, in front of our eyes …

Straightening the sheets.

‘She’s a famous literary critic you know. In the newspapers and everything. Must be one heck of a smart lady.’

Another: ‘What is the time, my love? Can you guess?’

Not quite.

‘Do you know what year we’re in?’

Time. Date.

… Reminiscence Therapy, there’s a decent evidence base in dementia. Reliving the sentiments of the past. Nostalgia as medication … eventually they lose the capacity for mental time travel, the vividness of a life lived fades into the wash of time, but until then …

What is there outside of our time? Beyond our clocks, calendars, years?

Nothing? Or perhaps cycles, repetitions, recapitulations. The border between past and future traversed by a meandering stream. Paths of duration forking like cracks in the ice.

Where is he? Mike?

Time existing only as memories. As remembered stories.

The stream is drying up.

‘Michael? He passed away a few years back my love, don’t you remember? There’s a lovely photograph of you two together, in the Alps or someplace like that. Let me fetch it for you.’

Shuffling a fresh deck of cards increases its entropy, but only by the criteria by which we define order. Clubs, diamonds, hearts, spades. Ace high, two low.

But what if there were other, different, ways of defining order and chaos? What if we found order *in* chaos?

Then, what might time be like?

She decides to experiment; become a scientist, like him. A traveller in time.

She first searches the present, but he cannot be found here. So, she must go backwards. The deck is shuffled. She bends time’s arrow, shooting in reverse, stretching herself away from the present. Her searchlight bursts through a prism, sending spectra into space. She is drawn to him like a magnet, his force is inescapable. She slings into the emptiness of the cosmos.

… drifting …

… alone …

… she tunnels through wormholes …

… burrows into the recesses of spacetime …

… then, finally, she enters his orbit, feeling the unmistakable pull of his gravity. The dim, lonely present recedes further into the distance. It is darker here, but a twinkle of his matter shines somewhere in the distance.

And then she finds him.

She finds him, deep in the black hole of time, waiting there as he said he would, where he always was, where he will always be. Never to escape from her again. They entangle, two quanta amidst a vastness which once separated them.

Through the throes of infinity, he speaks to her. Ahh, she knows these words well. He remembers, Jorge Luis Borges, her favourite author:

Time is a river which sweeps me along, but I am the river; it is a tiger which destroys me, but I am the tiger; it is a fire which consumes me, but I am the fire. Time is the substance I am made of.

Time is forever the substance they are made of.

The clock on the wall ticks over to the hour. Winter sun streams through a bay window, panes frosted by the first snow. A young woman wipes down the mattress, royal blue to match her frock. She carefully lays out the sheets and duvet. A sanitized smell hangs in the air.

Down the hallway, a telephone rings. The woman drops a battered deck of playing cards into a box on the way out.

‘Good morning, Berghof Nursing Home, Sister speaking.’

….

‘Yes, the ambulance came and took her early this morning … we’re packing away her things now …’

As she speaks, she turns over the picture frame to get a closer look. In the photograph they are draped together, puffer jackets zipped, cheeks aglow with the warmth of their love. She shudders, is choked.

What is left when the memories have gone? She thinks.

What is left?

## Figures and Tables

**Figure:**
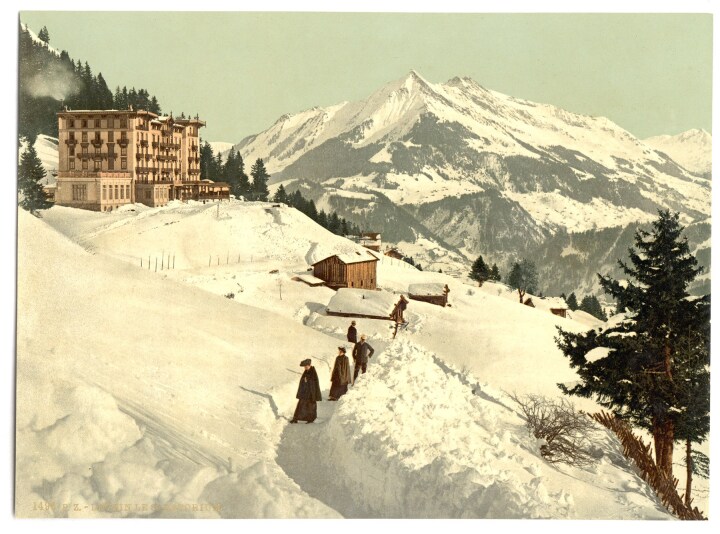
Image: The Magic Mountain. Photochrom Print Collection—Library of Congress Catalog: http://lccn.loc.gov/2001703352; in the Public Domain https://commons.wikimedia.org/w/index.php?curid=33176018

